# Supplementary material for: The Systems Biology Research Tool: evolvable open-source software
Source: BMC Syst Biol. 2008 Jun 29;2:55. doi: 10.1186/1752-0509-2-55 (PMC2446383; doi:10.1186/1752-0509-2-55)
Supplement: Additional file 1 — SBRT Archive. An archive of the current version of the Systems Biology Research Tool. [file 1752-0509-2-55-S1.zip › sbrt-1.4.0/doc/developers_guide/api/sbrt/shell/text/NonEmptyStringFormat.html]

NonEmptyStringFormat


|  |  |  |
| --- | --- | --- |
| **Overview** \| **Package** \| **Class** \| **Use** \| **Tree** \| **Deprecated** \| **Index** \| **Help** | |  |
| **PREV CLASS**   **NEXT CLASS** | **FRAMES**    **NO FRAMES**     **All Classes** |  |
| SUMMARY: NESTED \| FIELD \| CONSTR \| METHOD | DETAIL: FIELD \| CONSTR \| METHOD |  |


---


## sbrt.shell.text Class NonEmptyStringFormat

```
java.lang.Object
  sbrt.shell.text.NonEmptyStringFormat
```

**All Implemented Interfaces:**: Format, Formatter<java.lang.String>, Parser<java.lang.String>, SimpleFormat<java.lang.String>

---

``` public final class NonEmptyStringFormat extends java.lang.Object implements SimpleFormat<java.lang.String> ```

This class is used to define a format for `String`
objects.
Since `String` objects are already formatted
as strings, no formatting or parsing is actually required.
This class can be thought of as the trivial format, except that
non-empty strings are not allowed.

**Author:**
:   This class was written and documented by
    Jeremiah Wright while in the Wagner lab.

---

**Constructor Summary**

`NonEmptyStringFormat()`


| **Method Summary** | |
| --- | --- |
| `java.lang.String` | `format(java.lang.String obj)`             Returns the provided string. |
| `java.lang.String` | `parse(java.lang.String s)`             Returns a string equal to the one provided. |

**Methods inherited from class java.lang.Object**

`clone, equals, finalize, getClass, hashCode, notify, notifyAll, toString, wait, wait, wait`

**Constructor Detail**

### NonEmptyStringFormat

```
public NonEmptyStringFormat()
```


**Method Detail**

### format

```
public java.lang.String format(java.lang.String obj)
```

:   Returns the provided string.

    :   **Specified by:**: `format` in interface `Formatter<java.lang.String>`
    :   **Parameters:**: `obj` - the string that is returned. **Returns:**: the provided string. **Throws:**: `java.lang.IllegalArgumentException` - if `obj` is empty.

---


### parse

```
public java.lang.String parse(java.lang.String s)
```

:   Returns a string equal to the one provided.

    :   **Specified by:**: `parse` in interface `Parser<java.lang.String>`
    :   **Parameters:**: `s` - the string that is returned. **Returns:**: a string equal to the one provided. **Throws:**: `FormatException` - if `s` is empty.


---


|  |  |  |
| --- | --- | --- |
| **Overview** \| **Package** \| **Class** \| **Use** \| **Tree** \| **Deprecated** \| **Index** \| **Help** | |  |
| **PREV CLASS**   **NEXT CLASS** | **FRAMES**    **NO FRAMES**     **All Classes** |  |
| SUMMARY: NESTED \| FIELD \| CONSTR \| METHOD | DETAIL: FIELD \| CONSTR \| METHOD |  |


---
